# High genital prevalence of cutaneous human papillomavirus DNA on male genital skin: the *HPV Infection in Men Study*

**DOI:** 10.1186/s12879-014-0677-y

**Published:** 2014-12-09

**Authors:** Laura Sichero, Christine M Pierce Campbell, William Fulp, Silvaneide Ferreira, João S Sobrinho, Maria Luiza Baggio, Lenice Galan, Roberto C Silva, Eduardo Lazcano-Ponce, Anna R Giuliano, Luisa L Villa, for the HIM Study group

**Affiliations:** Molecular Biology Laboratory, Center of Translational Oncology, Instituto do Câncer do Estado de São Paulo (ICESP), Av. Dr. Arnaldo, 251, 8 andar, 01246-000, Cerqueira César, São Paulo, SP Brazil; Center for Infection Research in Cancer, H. Lee Moffitt Cancer Center and Research Institute, Tampa, FL USA; Ludwig Institute for Cancer Research, São Paulo, Brazil; Centro de Referência e Treinamento DST/Aids, São Paulo, 04121-000 Brazil; Instituto Nacional de Salud Publica, Cuernavaca, Mexico; Department of Radiology and Oncology, School of Medicine, School of Medicine of the University of São Paulo and HPV Institute, Santa Casa de São Paulo, Brazil

**Keywords:** Human papillomavirus, Cutaneous HPV, Males, HIM Study, Prevalence, Prospective study

## Abstract

**Background:**

The genital skin of males hosts a diversity of HPV genotypes and uncharacterized HPV genotypes. Previously we demonstrated that a specific viral genotype was not identified in 14% of all genital specimens (i.e., HPV unclassified specimens) using the Roche Linear Array method. Our goal was to identify and assess the prevalence of individual HPV types among genital HPV unclassified specimens collected in the HIM Study population, at enrollment, and examine associations with socio-demographic and behavioral characteristics.

**Methods:**

Genital skin specimens of men that were considered unclassified (HPV PCR positive, no genotype specified) at enrollment were typed by sequencing amplified PGMY09/11 products or cloning of PGMY/GP+ nested amplicons followed by sequencing. PGMY/GP+ negative specimens were further analyzed using FAP primers. HPV type classification was conducted through comparisons with sequences in the GenBank database.

**Results:**

Readable nucleotide sequences were generated for the majority of previously unclassified specimens (66%), including both characterized (77%) and yet uncharacterized (23%) HPV types. Of the characterized HPV types, most (73%) were Beta [β]-HPVs, primarily from β-1 and β-2 species, followed by Alpha [α]-HPVs (20%). Smokers (current and former) were significantly more likely to have an α-HPV infection, compared with any other genus; no other factors were associated with specific HPV genera or specific β-HPV species.

**Conclusions:**

Male genital skin harbor a large number of β-HPV types. Knowledge concerning the prevalence of the diverse HPV types in the men genital is important to better understand the transmission of these viruses.

**Electronic supplementary material:**

The online version of this article (doi:10.1186/s12879-014-0677-y) contains supplementary material, which is available to authorized users.

## Background

Human papillomaviruses (HPVs) are a diverse group of circular double-strand DNA viruses of approximately 8.000pb. Persistent infection with high-risk HPV is not only the major etiological factor for cervical cancer development but also for a high proportion of tumors of the vagina, vulva, penis, anal canal and oropharynx [1].

A novel papillomavirus is defined whenever the complete *L1* sequence of a viral genome differs by at least 10% from that of all characterized types [2]. To date, more than 180 different HPV types have been fully sequenced and characterized. The majority of HPVs comprise three genera: Alpha [α]-, Beta [β]- and Gamma [γ]-papillomavirus. α-HPV types have been predominantly isolated from mucosal and genital lesions, and include the 13 viral types classified as high-risk oncogenic (carcinogen type I) by the World Health Organization due to their increased prevalence in cervical cancer. β- and γ-HPV types were mainly isolated from the skin and for this reason have been grouped together as cutaneous HPV. β-HPVs are additionally divided into 6 different species including 45 HPV types, of which β-1 and β-2 are the most diverse and prevalent. Similarly, γ-HPVs are separated into 20 species, which together include 50 different HPV types. Partial DNA sequence information originating from several studies points towards the existence of hundreds of putative novel HPV types of the β- and γ-HPV genera [3],[4]. Recent data describe high prevalence of cutaneous HPV types at body sites that are different from those in which they were originally isolated [5]-[8].

During the last decade, there has been growing interest in understanding HPV natural history and related diseases among men. The HPV Infection in Men (HIM) Study is an ongoing, prospective genital HPV natural history study of over 4.000 men aged 18–70 years residing in Brazil (São Paulo), Mexico (Cuernavaca), and the United States (USA; Tampa). In this study, over 66% of men tested positive for HPV at their first study visit; however, the viral type could not be identified in 14% of the genital specimens using the Linear Array method (i.e., HPV PCR positive, no genotype specified) [9]. Our goal was to examine the prevalence of HPV types among genital HPV unclassified specimens collected among HIM Study participants at enrollment, and examine associations with socio-demographic and behavioral characteristics.

## Methods

### Study population

Men were enrolled between 2005 and 2009 and reported no prior diagnosis of genital warts or anogenital cancers, and had no recent symptoms of or treatment for a sexually transmitted infection, including HIV/AIDS. Men completed a pre-enrollment (baseline) visit, were enrolled on completion of their second (enrollment) visit two weeks post-baseline, and subsequently followed approximately every six months for up to four years. Details of the HIM Study cohort are described elsewhere [10],[11]. The present cross-sectional prevalence study was conducted among the first 3,105 men who completed their enrollment visits between July 2005 and December 2008. The IRD of the University of South Florida, Tampa, USA, the Instituto Mexicano del Seguro Social, and the Instituto de Salud Publica de Mexico, Mexico, and the Centro de Referência e Treinamento DST//Aids, Brazil, approved all study procedures, and all participants provided written informed consent.

### Genital specimen collection, DNA extraction, and HPV detection

At each study visit, participants completed a risk factor questionnaire and underwent a clinical examination of the external genital skin. Exfoliated cells were obtained from the coronal sulcus/glans penis, penile shaft, and scrotum using Dacron swabs (Digene, Gaithersburg, MD, USA), and these samples were further combined and stored at −80°C. HPV DNA was extracted using the QIAamp Media MDx Kit (Qiagen, Valencia, CA, USA). Samples were PCR amplified using PGMY09/11 generic primers and genotyped using the Roche Linear Array (Roche Molecular Diagnostics, Alameda, CA, USA). This technique allows testing for the presence of 37 α-HPV types commonly detected at the cervix (high-risk: 16, 18, 31, 33, 35, 39, 45, 51, 52, 56, 58, 59, 68; low-risk: 6, 11, 26, 40, 42, 53, 54, 55, 61, 62, 64, 66, 67, 69, 70, 71, 72, 73, 81, 82, 82 subtype IS39, 83, 84, 89 [CP6108]) [12]. Samples that tested PCR-positive and Linear Array-negative were considered unclassified and underwent additional typing.

### Typing of unclassified samples

Initially, purified DNAs were HPV genotyped by direct sequencing of PGMY09/11 primers PCR amplimers or cloning of these amplicons followed by sequencing. Next, 1 μl of PGMY09/11 negative product was used in a nested PCR using GP5+/6+ primers [13] and positive samples were cloned and sequenced. Finally, nested PGMY09/11-GP5+/6+ PCR negative samples were submitted to a novel amplification reaction employing FAP59/64 primers [14], and amplimers were analyzed solely by direct sequencing. All PCRs were performed using AmpliTaq Gold polymerase (Perkin-Elmer, Foster City, CA, USA). Before direct sequencing, PCR products were purified using the EXO SAP-IT (GE Healthcare, Buckinghamshire, UK). Sequencing reactions were performed in an ABI 3130XL Genetic Analyzer (AB Applied Biosystems, CA, USA) using the BigDye Terminator v3.1 Cycle Sequencing kit (AB Applied Biosystems, CA, USA). Sequence identity was determined by comparison with the BlastN database; sequences with identity scores higher than 90% within at least 200 bp were conclusively typed.

### Statistical analysis

The prevalence and genotype-distribution of unclassified genital HPV infections were estimated among all men enrolled in the HIM Study before December 2008. Infections were considered HPV-negative, HPV-positive for a characterized type, HPV-positive for an uncharacterized type, or untyped (i.e., inconclusive). Characterized HPV types were further classified according to genus and species. Socio-demographic and behavioral risk factors thought to be associated with presence of 1) a specific genus (α-, β-, γ-, or other HPV types), and 2) a specific β-HPV species were evaluated using exact Pearson chi-square tests and Monte Carlo methods. All statistical tests were two-sided and attained statistical significance at α = 0.05. Analyses were performed using SAS version 9.3 (SAS Institute, Cary, NC, USA).

## Results

Of the 3105 men enrolled in the HIM Study through December 2008, 1126 (36.3%) were HPV negative at their enrollment visit, 1572 (50.6%) had at least one of the 37 α-HPV types detected in the Linear Array assay, and 407 (13.1%) had an unclassified HPV type detected. Of the 407 samples with unclassified HPV at enrollment, 404 were available for additional characterization. These include 165, 134, and 105 men from the USA, Brazil and Mexico, respectively. Table 1 presents socio-demographic characteristics of participants included in this analysis, by country. Men with unclassified HPV were more likely to be younger (18–30 years, 52.5%), white (54.7%), non-Hispanic (63.0%), single (52.2%), uncircumcised (56.7%), never smokers (66.2%), moderate drinkers (41.6 and men who have sex with women (89.2%).

**Table 1:** **Characteristics of 404 participants from the** ***HIM Study*** **with unclassified HPV in the genital area at enrollment**

	Total n = 404	USA n = 165	Brazil n = 134	Mexico n = 105	*P* value ^a^
Characteristic	n (%)	n (%)	n (%)	n (%)	
**Age, years**					<.001
**Median (range)**	30 (18–70)	20 (18–70)	36 (18–70)	35 (18–67)	
**Mean (SD)**	32.1 (12.5)	25.6 (11.5)	36.8 (11.0)	36.3 (11.4)	
**18–30**	212 (52.5%)	131 (79.4%)	43 (32.1%)	38 (36.2%)	
**31–44**	130 (32.2%)	21 (12.7%)	63 (47.0%)	46 (43.8%)	
**≥45**	62 (15.3%)	13 (7.9%)	28 (20.9%)	21 (20.0%)	
**Race**					<.001
**White**	221 (54.7%)	127 (77%)	87 (64.9%)	7 (6.7%)	
**Black**	48 (11.9%)	14 (8.5%)	34 (25.4%)	0 (0%)	
**Asian/Pacific Islander**	10 (2.5%)	10 (6.1%)	0 (0%)	0 (0%)	
**Mixed race**	122 (30.2%)	13 (7.9%)	12 (9.0%)	97 (92.4%)	
**Missing data**	3 (0.7%)	1 (0.6%)	1 (0.7%)	1 (1.0%)	
**Ethnicity**					<.001
**Non-Hispanic**	254 (63.0%)	142 (86.1%)	112 (84.2%)	0 (0%)	
**Hispanic**	149 (37.0%)	23 (13.9%)	21 (15.8%)	105 (100%)	
**Marital status**					<.001
**Single**	211 (52.2%)	139 (84.2%)	47 (35.1%)	25 (23.8%)	
**Married**	131 (32.4%)	15 (9.1%)	56 (41.8%)	60 (57.1%)	
**Cohabitating**	45 (11.1%)	4 (2.4%)	24 (17.9%)	17 (16.2%)	
**Divorced, separated, or widowed**	17 (4.2%)	7 (4.2%)	7 (5.2%)	3 (2.9%)	
**Education, years**					<.001
**<12**	76 (18.9%)	0 (0%)	35 (26.3%)	41 (39.0%)	
**12**	100 (24.8%)	30 (18.2%)	52 (39.1	18 (17.1%)	
**13–15**	146 (36.2%)	110 (66.7%)	20 (15.0%)	16 (15.2%)	
**16**	60 (14.9%)	16 (9.7%)	22 (16.5%)	22 (21.0%)	
**≥17**	21 (5.2%)	9 (5.5%)	4 (3.0%)	8 (7.6%)	
**Circumcision**					<.001
**No**	229 (56.7%)	36 (21.8%)	111 (82.8%)	82 (78.1%)	
**Yes**	175 (43.3%)	129 (78.2%)	23 (17.2%)	23 (21.9%)	
**Smoking status**					<.001
**Never**	266 (66.2%)	116 (70.7%)	97 (72.9%)	53 (50.5%)	
**Former**	73 (18.2%)	19 (11.6%)	26 (19.5%)	28 (26.7%)	
**Current**	63 (15.7%)	29 (17.7%)	10 (7.5%)	24 (22.9%)	
**Alcohol intake, drinks per month**					<.001
**0**	106 (27.0%)	29 (17.9%)	52 (41.3%)	25 (24.0%)	
**1–30**	163 (41.6%)	53 (32.7%)	52 (41.3%)	58 (55.8%)	
**≥31**	123 (31.4%)	80 (49.4%)	22 (17.5%)	21 (20.2%)	
**Sexual orientation**					0.006
**MSW**	337 (89.2%)	145 (93.5%)	102 (81%)	90 (92.8%)	
**MSM**	22 (5.8%)	7 (4.5%)	12 (9.5%)	3 (3.1%)	
**MSWM**	19 (5.0%)	3 (1.9%)	12 (9.5%)	4 (4.1%)	
**Lifetime number of sex partners**					0.003
**0–2**	132 (33.0%)	59 (36.2%)	40 (30.3%)	33 (31.4%)	
**3–7**	118 (29.5%)	53 (32.5%)	25 (18.9%)	40 (38.1%)	
**8–19**	90 (22.5%)	27 (16.6%)	40 (30.3%)	23 (21.9%)	
**≥20**	60 (15.0%)	24 (14.7%)	27 (20.5%)	9 (8.6%)	
**Diagnosis of sexually transmitted infection, ever**					0.003
**No**	344 (85.6%)	152 (92.7%)	107 (80.5%)	85 (81.0%)	
**Yes**	58 (14.4%)	12 (7.3%)	26 (19.5%)	20 (19.0%)	

MSW, men who have sex with women; MSM, men who have sex with men; MSWM, men who have sex with women and men. ^a^Pearson chi-square *P* value using exact Monte Carlo methods.

HPV type distribution of previously unclassified HPV infections is shown in Table 2. HPV could not be genotyped among 121 (30.0%) FAP59/64 PCR-positive specimens; direct sequencing was inconclusive due to overlapping peak patterns. Among 265 (65.6 specimens with successful sequences, 60 (22.6%) were considered uncharacterized; sequence comparison analysis showed high identity to partial HPV nucleotide sequences in GenBank without type designation at this time. Among the other 205 (77.4%) specimens with already characterized HPV types, 41 (20.0%) belonged to the α-HPV genus, 150 (73.2%) to the β-HPV genus, and 14 (6.8%) to the γ-HPV genus. The distribution of HPV types for each genus was similar across countries, though a higher proportion of γ-HPV types were detected in Brazil and Mexico as compared to the USA.

**Table 2:** **HPV type distribution of previously unclassified HPV infections, by country**

	Total n = 404	USA n = 165	Brazil n = 134	Mexico n = 105
	n (%)	n (%)	n (%)	n (%)
**HPV-negative**	18 (4.5)	5 (3.0)	7 (5.2)	6 (5.7)
**inconclusive HPV** ^**a**^	121 (30.0)	45 (27.3)	48 (35.8)	28 (26.7)
**Any HPV type** ^**b**^	265 (65.6)	115 (69.7)	79 (59.0)	71 (67.6)
**Uncharacterized HPV type**	60 (22.6)	30 (26.1)	15 (19.0)	15 (21.1)
**Characterized HPV type**	205 (77.4)	85 (73.9)	64 (81.0)	56 (78.9)
**Alpha (α)-HPV**	41 (20.0)	19 (22.4)	12 (18.8)	10 (17.9)
**Beta (β)-HPV**	150 (73.2)	63 (74.1)	46 (71.9)	41 (73.2)
**Gamma (γ)-HPV**	14 (6.8)	3 (3.5)	6 (9.4)	5 (8.9)

^a^sequences could not be interpreted.

^b^Characterized and uncharacterized types.

**Figure 1:**
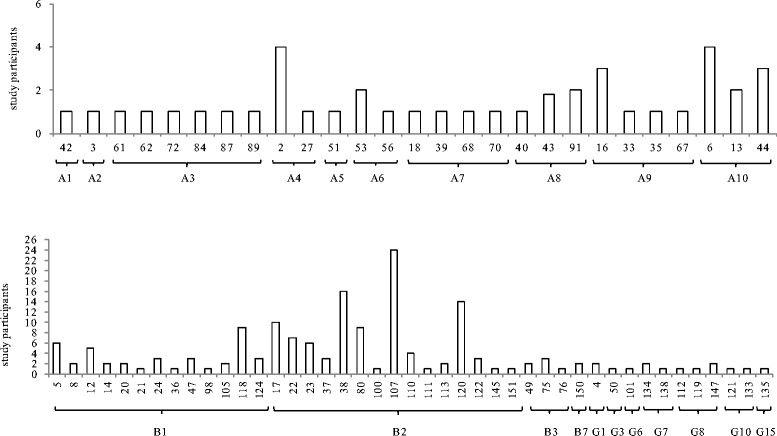
**HPV type distribution by HPV genus and types among unclassified genital samples of the HIM study at enrollment.** HPV types and species are indicated along the x-axis (n = 205).

The genotype-specific distribution of characterized HPV types is found in Figure 1. A total of 27 α-HPV types, 32 β-HPV types, and 14 γ-HPV types were detected. The most common α-HPV types included HPV-2 and HPV-6 (2.0 4 each), the most common β-HPVs included HPV-107 (11.7%), HPV-38 (7.8%), HPV-120 (6.8%), and HPV-17 (4.8%), and the most common γ-HPVs included HPV-4, HPV-134 and HPV-147 (1.0%, 2 each).

Of the risk factors, only cigarette smoking was significantly associated with detection of α-, β-, or γ-HPV types (Table 3); smokers (current and former) were significantly more likely to have an α-HPV infection, compared with any other genus (p = 0.039). No other factors were significantly associated with the detection of a specific HPV genus. Nevertheless, the prevalence of α-HPV types was non-significantly highest among participants living in the USA, and those aged 18–30 years, white, non-Hispanic, single, circumcised, and reported ≥8 lifetime sex partners. In contrast, the prevalence of β-HPV types was equally as high among men who were single and married/cohabitating, and highest among men who were uncircumcised and reported <8 lifetime sex partners. γ-HPV types were more common in Brazil and Mexico than USA, and among men who were married/cohabitating, uncircumcised, and reported <8 lifetime sex partners. Further, none of the risk factors examined were significantly associated with particular β-HPV species detection.

**Table 3:** **Factors associated with HPV infections by genus in the male genital area**

Characteristic	Total n = 265 ^a^	Alpha n = 41	Beta n = 150	Gamma n = 14	Uncharacterized n = 60	*P* value ^b^
	n (%)	n (%)	n (%)	n (%)	n (%)	
Country						0.649
USA	115 (43.4)	19 (46.3)	63 (42.0)	3 (21.4)	30 (50.0)	
Brazil	79 (29.8)	12 (29.3)	46 (30.7)	6 (42.9)	15 (25.0)	
Mexico	71 (26.8)	10 (24.4)	41 (27.3)	5 (35.7)	15 (25.0)	
Age, years						0.279
18–30	130 (49.1)	23 (56.1)	75 (50.0)	7 (50.0)	25 (41.7)	
31–44	89 (33.6)	9 (22.0)	49 (32.7)	7 (50.0)	24 (40.0)	
≥45	46 (17.4)	9 (22.0)	26 (17.3)	0 (0)	11 (18.3)	
Race						0.492
White	146 (55.1)	21 (51.2)	81 (54.0)	6 (42.9)	38 (63.3)	
Black	30 (11.3)	9 (22.0)	15 (10.0)	1 (7.1)	5 (8.3)	
Asian/Pacific Islander	7 (2.6)	1 (2.4)	4 (2.7)	0 (0)	2 (3.3)	
Mixed race	80 (30.2)	10 (24.4)	48 (32.0)	7 (50.0)	15 (25.0)	
Missing data	2 (0.8)	0 (0)	2 (1.3)	0 (0)	0 (0)	
Ethnicity						0.503
Non-Hispanic	168 (63.6)	29 (70.7)	90 (60.0)	8 (61.5)	41 (68.3)	
Hispanic	96 (36.4)	12 (29.3)	60 (40.0)	5 (38.5)	19 (31.7)	
Marital status						0.328
Single	132 (49.8)	26 (63.4)	72 (48.0)	4 (28.6)	30 (50.0)	
Married or cohabitating	121 (45.7)	14 (34.1)	69 (46.0)	10 (71.4)	28 (46.7)	
Divorced, separated, or widowed	12 (4.5)	1 (2.4)	9 (6.0)	0 (0)	2 (3.3)	
Education, years						0.761
≤12	116 (43.9)	18 (43.9)	65 (43.3)	6 (46.2)	27 (45.0)	
13–15	96 (36.4)	15 (36.6)	53 (35.3)	3 (23.1)	25 (41.7)	
≥16	52 (19.7)	8 (19.5)	32 (21.3)	4 (30.8)	8 (13.3)	
Circumcision						0.470
No	135 (50.9)	17 (41.5%)	77 (51.3)	9 (64.3)	32 (53.3)	
Yes	130 (49.1)	24 (58.5%)	73 (48.7)	5 (35.7)	28 (46.7)	
Smoking status						0.039
Never	177 (67.3)	23 (56.1)	100 (67.1)	12 (92.3)	42 (70.0)	
Former	45 (17.1)	6 (14.6)	31 (20.8)	1 (7.7)	7 (11.7)	
Current	41 (15.6)	12 (29.3)	18 (12.1)	0 (0)	11 (18.3)	
Alcohol intake, drinks per month						0.532
0	67 (26.2)	8 (20.5)	38 (26.2)	5 (41.7)	16 (26.7)	
1–30	109 (42.6)	17 (43.6)	66 (45.5)	5 (41.7)	21 (35.0)	
≥31	80 (31.3)	14 (35.9)	41 (28.3)	2 (16.7)	23 (38.3)	
Sexual orientation						0.761
MSW	223 (90.3)	31 (83.8)	125 (90.6)	13 (100)	54 (91.5)	
MSM	13 (5.3)	3 (8.1)	7 (5.1)	0 (0)	3 (5.1)	
MSWM	11 (4.5)	3 (8.1)	6 (4.3)	0 (0)	2 (3.4)	
Lifetime number of sex partners						0.152
0–2	77 (29.5)	9 (22.0)	49 (33.1)	6 (46.2)	13 (22.0)	
3–7	85 (32.6)	9 (22.0)	49 (33.1)	4 (30.8)	23 (39.0)	
8–19	58 (22.2)	15 (36.6)	27 (18.2)	3 (23.1)	13 (22.0)	
≥20	41 (15.7)	8 (19.5)	23 (15.5)	0 (0)	10 (16.9)	
Diagnosis of sexually transmitted infection, ever						0.887
No	230 (87.5)	35 (85.4)	131 (87.3)	11 (84.6)	53 (89.8)	
Yes	33 (12.5)	6 (14.6)	19 (12.7)	2 (15.4)	6 (10.2)	

^a^Total number of men with characterized (α-, β- or γ-HPV) or uncharacterized HPV types.

^b^Pearson chi-square *P* value using exact Monte Carlo methods.

## Discussion

Recently we reported our initial observations concerning the broad distribution of β- and γ-HPV types in genital specimens of men participating in the HIM Study at enrollment through three years of follow-up [8]. We then analyzed the β-HPV type distribution in external genital lesion (EGL) specimens and preceding normal genital skin specimens among HIM participants who developed external genital lesions [7]. In the present study, we expand our previous work by presenting viral sequence characterization of all unclassified HPV genital specimens at the time of participant enrollment and by evaluating the association of HPV detection with participant characteristics and sexual behavior. Some studies have reported the presence of cutaneous HPV types in penile carcinomas [15] and in cervical and penile condylomas [16],[17]. However, to our knowledge the HIM study is unique in searching for cutaneous HPV in normal male genital skin specimens.

Among the 404 samples analyzed, 4.5% were truly HPV negative, i.e. PCR negative using any of the three primers sets, suggesting that a small proportion of unclassified HPV detection presumably represents spurious amplification. In 30.0% of the specimens, unreadable sequences were generated after direct sequencing of FAP54/69 PCR products; among these the presence of more than one HPV type is suspected [14]. This hypothesis has been confirmed through sequencing of a number of clones from each sample whenever overlapping peak patterns were observed and also by the Luminex technology [7],[8]. DNA from multiple cutaneous HPV types has been commonly detected not only in the normal skin and cutaneous tumors [16],[18],[19] but also in oral cavity specimens [5], and in the anogenital tract [8]. Together these data suggest that cutaneous HPV types may be a commensal component of the microbiological flora of the human skin [4],[20]-[22]. We also observed that nucleotide sequences of 60 specimens mostly consisted of partial FAP59/64 sequences previously deposited in the GenBank database [23],[24], and were therefore considered uncharacterized HPV types. Currently, more than 200 different putative HPV types are pending full genome characterization, with the majority clustering within the γ-HPV genus [3]. We detected several α-HPVs that should have been identified with the Linear-array genotyping test. These false-negative results are likely due to low viral copy numbers which were detected on a different PCR reaction followed by sequencing, a more sensitive technical approach.

Overall, unclassified HPV detection in HIM participants was significantly associated with younger age, white race, and non-Hispanic ethnicity. β-HPV types were detected among individuals residing in three countries and with diverse habits; however, slight differences in participant characteristics were demonstrated in men with α-HPVs versus β-HPVs, though none of these differences achieved statistical significance. In contrast to α-HPV prevalence in the genital region of men from the HIM study [25], β-HPV DNA detection was not significantly associated with sexual behaviors, indicating that viral types from this genus may not be transmitted sexually. The risk factor questionnaire completed by HIM participants focuses mostly on penetrative sexual behavior and lacks information on masturbation practices and non-penetrative sexual activities, which may be helpful in understanding the mechanism of transmission for this group of viruses. It is plausible that direct hand-to-penis contact may favor viral transmission of cutaneous HPV to the genital area. In fact, evidence of hand transmission to the anogenital region has been reported for α-HPV types [26]. Furthermore, α-HPVs have been detected on fingers of women who are infected at the cervix [27]. Nevertheless, the possibility remains that the detection of β-HPVs in male genitals may also represent deposition of virions shed from other body sites with productive infections.

HPV characterization of skin specimens from healthy individuals and squamous cell carcinomas of the skin (SCC) indicated that HPV types of β-2 species predominates in tumor samples [24]. In the present study, the analysis of participant characteristics and sexual behavior stratified by β-HPV species found no significant associations. As with all studies, there are limitations that could influence the interpretation of the results obtained. This study was conceived to identify HPV types that were grouped together as unclassified HPV types at enrollment in the HIM Study. For this reason, this analysis excluded specimens for which an HPV type was already assigned by Linear Array, although these specimens may have also harbored β- and γ-HPV types. Co-detection with α- and β-HPV types was shown to be common in the oral cavity [5] and in penile condylomas [28].

The role of HPV in the development of non-melanoma skin cancer is not well defined. Furthermore, it is unknown whether HPV DNA detected in healthy skin represents viral particles or superficial cells containing episomal HPV genomes [29]. It has been suggested that skin infected with β-HPV types harbor increased susceptibility to ultraviolet radiation induced DNA damage, ultimately resulting in skin cancer [30]. At present, it is consensus that HPV-5 and HPV-8 are associated with benign and malignant lesions of the cutaneous disease *epidermodysplasia verruciformis* [31]. Recent data show the presence of β-HPV types in the oral cavity [5],[32], cervical and penile condylomas [17],[33] and also in cervicovaginal cells [6],[34]. Nevertheless, at least for the oral cavity, only viral transcripts of α-HPV were detected in non-malignant lesions and cancer co-harboring β- or γ-HPV DNA [35]. Most studies examining the role of HPV in the development of male EGL have evaluated mucosal HPV types of the α-HPV genus. We observed that β-HPV DNA is prevalent in EGLs (61.1%) and that most viral DNAs are detected on the normal genital skin before EGL development [7]. At present it is unclear how β-HPV types may influence lesion development in the genital region of men. Although detection of cutaneous HPV types in the genital region may simply reflect virions released from other parts of the body once β- and γ-HPV are ubiquitous throughout the body, a possible role for these viruses as co-factors in the oncogenesis of penile cancer remains to be clarified. Studies conducted prospectively in a larger number of patients will contribute to this knowledge.

## Conclusions

Detection of HPV types not previously assigned by Roche´s Linear-Array in the genitals of participants of a natural history study of HPV in men was significantly associated with younger age, white race, and non-Hispanic ethnicity. Among these HPVs, we detected a broad range of α- and β-HPVs. Further research is needed to determine how these cutaneous HPV are transmitted.

### Ethical approval

Ethical approval was given by the University of South Florida (IRB# 102660), Ludwig Institute for Cancer Research, Centro de Referência e Treinamento em Doenças Sexualmente Transmissíveis e AIDS, and Instituto Nacional de Salud Pública de México.

## Authors’ original submitted files for images

Below are the links to the authors’ original submitted files for images. Authors’ original file for figure 1

## Abbreviations

HPV: Human papillomavirus
HIM Study: HPV Infection in Men Study
β-HPV: beta-HPV
α-HPV: alpha-HPV
γ-HPV: gamma-HPV
