# Supplementary figures and images for: High genital prevalence of cutaneous human papillomavirus DNA on male genital skin: the HPV Infection in Men Study
Source: BMC Infect Dis. 2014 Dec 9;14:677. doi: 10.1186/s12879-014-0677-y (PMC4265346; doi:10.1186/s12879-014-0677-y)

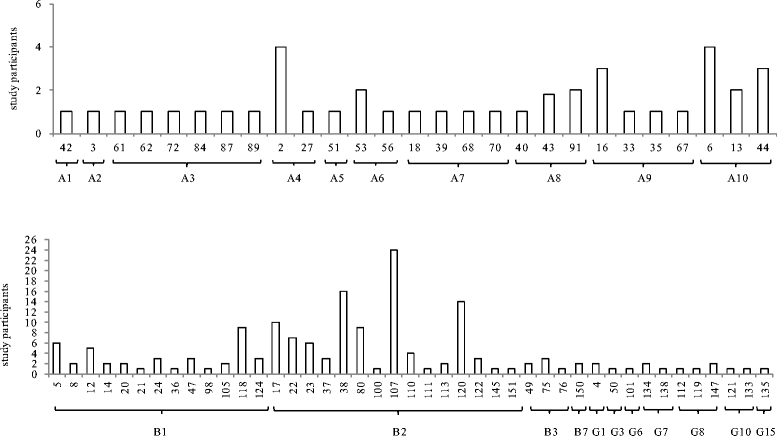

Supplement: Supplementary file 1 — Authors’ original file for figure 1 [file 12879_2014_677_MOESM1_ESM.gif]
